# Eleven quick tips for properly handling tabular data

**DOI:** 10.1371/journal.pcbi.1012604

**Published:** 2024-11-27

**Authors:** Marla I. Hertz, Ashley S. McNeill

**Affiliations:** University of Alabama at Birmingham Libraries, Birmingham, Alabama, United States of America; Origin Bioinformatics, CANADA

## Overview

Proper handling of tabular data, i.e., spreadsheets, is an essential skill for most researchers. Well-curated tabular data has built-in quality control, is machine readable by downstream analysis applications, and ultimately saves researchers’ time. The increased expectation of data sharing grounded by funder and publisher policies has made it even more crucial to format and describe tabular data properly to ensure that it is FAIR—findable, accessible, interoperable, and reusable [1]. However, many researchers are not formally trained in the data management skills necessary to build strong tabular datasets. They may also struggle to anticipate the future needs and potential applications of their data. This article attempts to demystify tabular data practices and outlines 11 tips presented roughly in the order of use during a research project. While these tips feature examples using the Microsoft Excel application, chosen deliberately because it is commonly used and is often the entry point software for data analysis, they are applicable to similar spreadsheet software programs.

## Introduction

Excel is a common entry point for working with tabular data despite the software not having robust fail-safes to guard data integrity. Errors in tabular data are pervasive and can have serious scientific and societal impacts. For example, faulty spreadsheet practices have led to errors in gene names [2,3], lost COVID-19 cases [4], and one study on healthcare in Ireland found that over 90% of the spreadsheets they examined were faulty [5].

**Fig 1:**
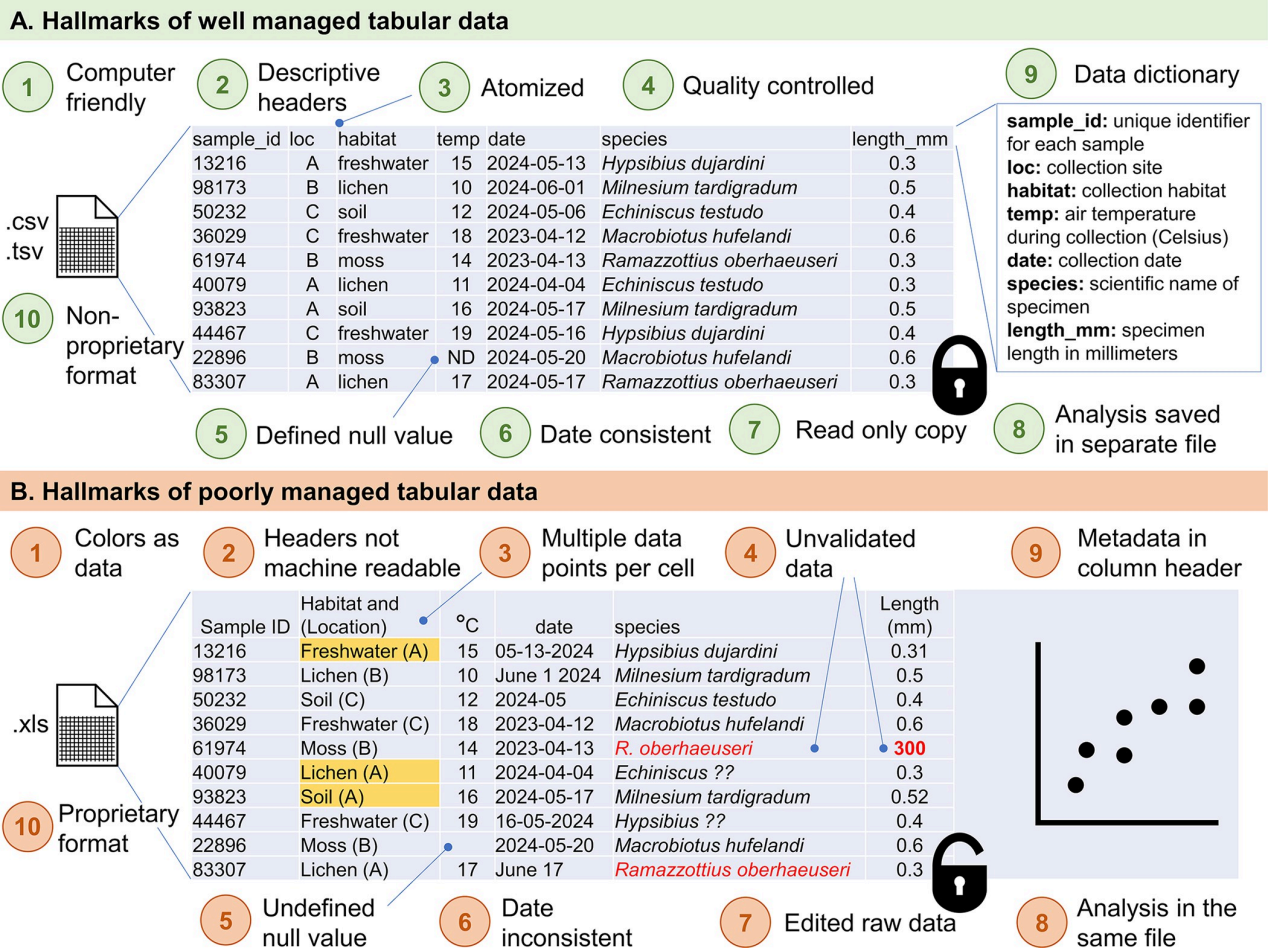
An illustration of the 11 tips for tabular data showcases good practices in green and poor practices in orange for tips 1 through 10. Tip 11 is not pictured. The sample data table includes synthetic data produced using ChatGPT 3.5 on 2024-07-31 using the following prompt: “generate a synthetic dataset about tardigrades that shows good data management, no merged cells, no color used as format, descriptive headers, computer friendly.” The ChatGPT output (location, habitat, species name, and length columns) was exported to Excel and modified slightly for machine readability. Sample identification (ID) numbers and collection dates were randomly generated in Excel.

Furthermore, as a researcher progresses in their career, it is assumed that they have the skills to manage increasingly complex data using spreadsheet software even though most researchers are not typically trained on how to use these program effectively. This gap has been addressed in other works not explicitly focused on Excel software [6,7]. This article provides guidelines for proper data management in Excel and tips to avoid common pitfalls (see Fig 1 for an illustrated outline of the tips). We also use this opportunity to weave in important data management concepts to conserve raw data, preserve final outputs, and document processes.

Of note, there are several 10 Simple Rules articles which complement our tips including rules for data management planning [8], maximizing the recommendations of the NIH data management and sharing plan [9], general data management [10], data storage [11], data sharing [12], and implementing FAIR data practices at the community level [13]. Importantly, this article will focus on small-scale data tables and is not meant to be a primer for big data management, which has been covered elsewhere [14,15]. Another Tips article covers numerical data more generally from data acquisition to dissemination of results [16]. Finally, a word of caution—these tips were based on the Microsoft 365 Excel accessed in 2024 (Version 2308 Build 16.0.16731.20542). Your copy of Excel may be different. Similar functions for quality control can be found in most comparable spreadsheet software.

## Tip 1: Format for computer readability, not human

When collecting data, it can be difficult to anticipate exactly how the data will be analyzed or presented in the future. The goal of creating raw tabular data is to capture as much detail about the work as possible to examine and draw conclusions. Yet, in attempting to make data easier for a person to digest, researchers may manipulate tabular data files in ways that hinder future analysis such as merging or color-coding cells. To make things easier for your future self and for others who might use your data, it is important to enter raw data in a computer readable format. Prioritizing computer-friendly formats over human-friendly ones has many benefits. For example, you or others may need to import that data into a program for analysis or make quick plots of the data to visualize relationships between variables, and these actions would be hindered by color-coded or merged cells.

How can you make sure your data is computer readable? For raw data files, you should never merge cells in a spreadsheet. When exporting and importing tabular data, rows and columns with different sizes—in other words, with inconsistent merged cells—can lead to errors in the computer’s interpretation of how the data is positioned, which can lead to mistakes in the computer’s interpretation of which data points belong together.

Another important aspect of collecting computer readable raw data is to always use a number or a string of symbols as individual datum. Using color as data (for example, the yellow-highlighted cells in Fig 1B) is often impossible to translate from one program to another. Color as data can also be misinterpreted by others interacting with your work—you may know what you mean by red or green, but others might have their own interpretations of this binary. It’s best to use “yes” or “no” instead; for numerical alternatives, consider 0 and 1. These values will be conserved between programs when data is transferred. The avoidance of color as data also makes it more accessible for those with color blindness. Thus, while practices to make an aesthetically appealing table are appropriate for preparing table as a data visualization for a presentation, they are to be avoided for raw data tables and at all steps in data analysis.

## Tip 2: Use descriptive single-row headers using machine-readable characters

**Fig 2:**
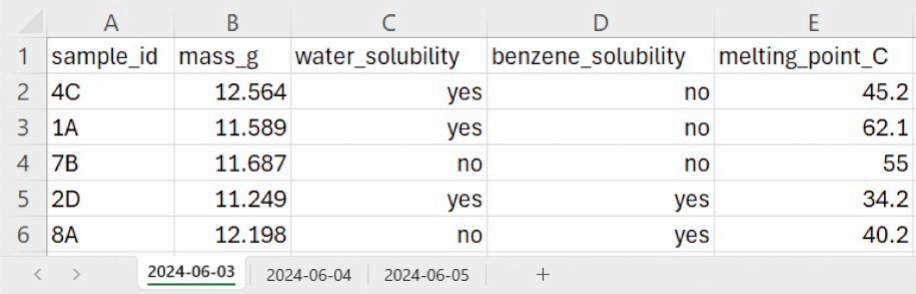
Example illustrating machine-readable spreadsheet header practices. Each spreadsheet tab is named according to the date the data was taken in YYYY-MM-DD format and header row labels are formatted without spaces, special characters, or unnecessary capital letters for computer readability. For use with other data analysis programs outside of Excel, these tabs should be saved as separate files with the date in the file name.

Most spreadsheet programs designate the first row of data as the header row for sorting data or as default labels in any chart or graph created with that data. Therefore, including multiple header rows can lead to errors when working with your spreadsheet. Especially for raw data files, limit to a single header row for each spreadsheet. It is often best practice to indicate units of measurement. In Fig 2, common shorthand for the units of mass and temperature are indicated in columns B and E. If more description about the data is needed, use a more specific file naming scheme. For example, if you are collecting the same types of data on 3 consecutive days, you could create 3 sheets within your spreadsheet file with the date the data was collected as the title of the spreadsheet (Fig 2). Be aware that with this method, each sheet will need to be saved separately if the data is converted to other file formats. Alternatively, include another column for the date that the data was collected. The article *Tidy Data* has more information on effective tabular data structures [17].

It is also important to carefully consider the column names within the header row. Each column should have short, descriptive, machine-readable names comprised of Arabic letters and numbers. Avoid using capital letters unless required to express units, such as “melting_point_C” in Fig 2 for degrees Celsius. It is also a best practice to never include spaces in the names of your files or variables, as demonstrated using underscores in the above example. If you are working in a line coding environment, spaces in file names can cause the files to be unreadable or lead to mistakes in processing. Avoid other special characters such as non-Arabic letters and punctuation, which can cause reading errors when data is imported into analysis codes or programs. For example, commas are particularly problematic when spreadsheets are saved in the comma separated value (.csv) file format.

## Tip 3: Atomize your data—One data point per cell

When creating raw data files, it is critically important to input only 1 piece of information in each cell of the spreadsheet. Ideally, each row of the spreadsheet will correspond to 1 sample or record and each column will correspond to a single variable or observation, as shown in Fig 2. This framework ultimately allows for greater flexibility when performing data analysis, such as data transformations. For example, in Fig 1B, the 2 variables habitat type (soil, lichen, etc.) and sample location (site A, B, or C) appear in a single column. These data should be separated into 2 columns as in Fig 1A to facilitate analysis by variable. It is also easier to select specific subsets of data to display for presentation when the data are arranged in separated columns. For example, in Fig 1A, we may rearrange the raw data columns to show the species column immediately after the sample ID, prioritizing the order of information based on how we anticipate our readers will access the content.

## Tip 4: Keep data consistent with quality control measures

Efforts to ensure data consistency and quality from the start will reduce the time spent tidying data prior to analysis. For example, in the poorly managed data in Fig 1B, the species name is inconsistent. Both *R*. *oberhaeuseri* and *Ramazzottius oberhaeuseri* are acceptable scientific nomenclature for the same organism; however, analysis software may incorrectly designate these as separate species and therefore this column needs to be standardized as in Fig 1A before analysis can begin.

**Fig 3:**
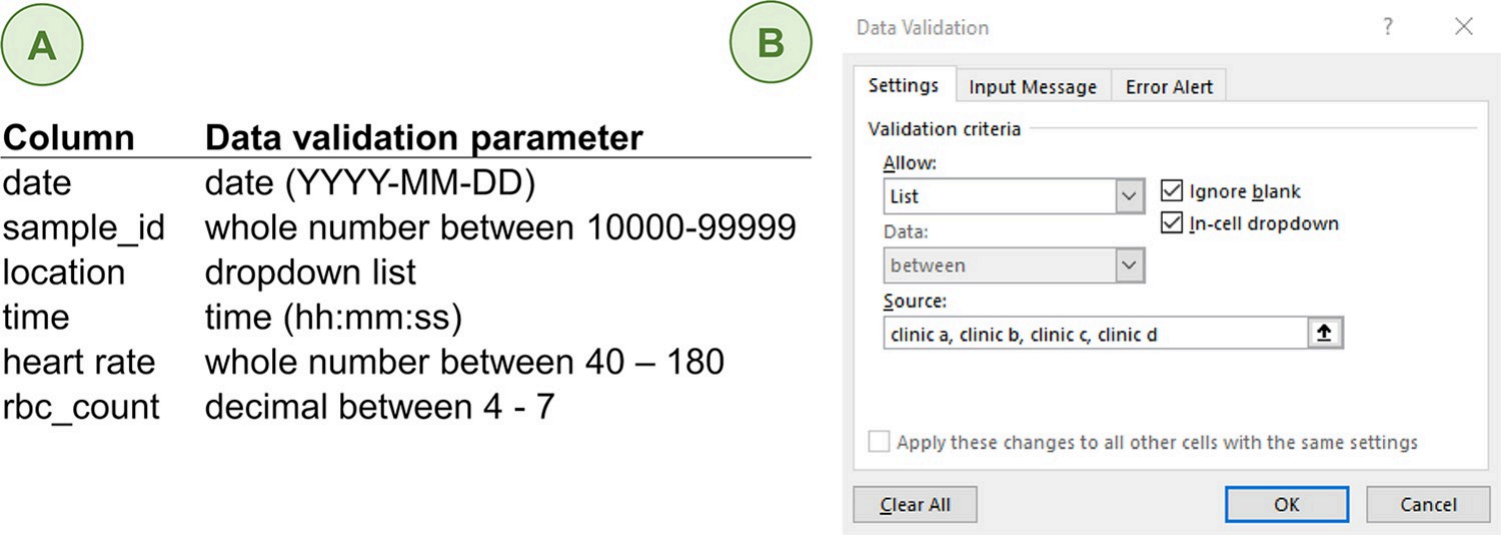
Quality control measures for a clinical dataset. **(A)** These parameters limit what can be entered in a cell to a range of acceptable values with standard formatting. RBC stands for red blood cell. **(B)** To set up data validation for each variable, go to the Data tab > Data Validation. Then, fill in the popup window with the data type allowed. The example shown here is for a dropdown list of location sites with the acceptable categories separated by commas.

The data validation feature in Excel is an underutilized but powerful tool for data quality. Have you ever entered a value into Excel with the decimal in the wrong place? Spreadsheets with data validation parameters set prior to data entry will block those types of mistakes. Consider the hypothetical clinical data in Fig 3A—each column/variable has a specific format and range of values. This information can be programmed into the spreadsheet so that only properly formatted values within the specified range can be entered into a cell. The steps necessary to set up data validation for each variable are shown in Fig 3B.

To minimize transcription errors when inputting data into spreadsheet software, design dropdown menus for categorical data to contain only allowed values. For example, if the purpose of the “location” column in Fig 3 is to document which clinic was used, limiting the answers to clinic A–D is prudent. Otherwise, this opens up interpretation to write other descriptors into this field such as the name of the clinic, the clinic letter only, or even GIS coordinates. Standardizing data input practices are especially critical when data tables are built over time and data entry is executed by more than 1 person.

Researchers also need to do a better job validating data prior to sharing. For example, as mentioned in the introduction, there is a pervasive problem in genetics research of published tabular data with dates in place of gene names because Excel automatically converts gene symbols to dates, such as SEPT1 (Septin 1) to September 1 of the current year. Therefore, it is incumbent upon the researcher to validate gene name entries prior to submitting their data for publication. Setting the format of the gene symbol column to “text” before data entry will prevent the conversion of gene names to dates. Additionally, geneticists have tackled this issue by renaming problematic gene symbols (e.g., SEPT1 became SEPTIN1) [18,19].

Other software is available to make data cleaning less onerous. The open-source software OpenRefine (RRID:SCR_021305) is adept at bulk tidying data sets to resolve inconsistencies. Alternatively, dedicated data entry software such as REDCap (RRID:SCR_003445) is a popular data entry tool for quality control especially in clinical disciplines. No matter which tool is used, incorporating data validation will make tabular data easier to use and harmonize with other datasets.

## Tip 5: Define null values

Another aspect that should be standardized and documented for each data set is how the table defines missing and null values. This will reduce ambiguity in the data set: Does a zero in the cell mean the measured value is zero or that data is missing? We recommend you review a summary table created by White and colleagues on how to address zero and null values based on which downstream application will be used [7]. Another related sign of untidy data is the presence of spaces before or after the data in the cell (also known as leading and trailing spaces respectively). Extra spaces are notoriously hard to detect in spreadsheet software which could invalidate analysis results. The OpenRefine tool mentioned in Tip 4 is adept at identifying and removing superfluous spaces.

## Tip 6: Exercise caution when working with dates and time as data

The international standard for date and time format, also known as ISO 8601, is to display the date as YYYY-MM-DD (for example, 2024-05-06) and the time as hours:minutes:seconds:milliseconds (for example, 13:01:00:03). If the seconds and milliseconds are not recorded or relevant, you can choose not to list them [20]. It can be worth mentioning in your data files whether the time recorded is in your local time zone or Coordinated Universal Time (UTC). While use of UTC may not be necessary for many projects, it is the generally accepted way to express time of day in a way that is not localized to the geographic location where the data was collected. It is also important to realize that “Day 1” in Excel is January 1, 1900. This is problematic for anyone doing historical research because any date prior to January 1, 1900, does not register as a date in Excel. Alternatively, Julian date (JD) is used in some fields to represent the date and time in a single value. The value that represents the current date and time is calculated by adding up the number of days since noon on January 1, 4713 BC [21].

Appropriate use of dates and time in tabular data can be a powerful tool for organization. Throughout a project, it is wise to periodically save a new file with the current date. This can help you keep track of versions of your work and can prevent major losses if one of your files becomes lost or corrupted. One way to do this is to add the date to the end of the file names in year-month-day format, such as experiment-1_cleaned-data_2024-05-06.csv. This will allow files to sort in chronological order when the folder is set to display alphabetically.

## Tip 7: Lock raw data files in a read-only format

This tip is often overlooked in the excitement to start analysis. Once the data collection phase is complete, stop and save raw data files separately in a read-only format. It is easy to unintentionally delete or modify cells in Excel. By having a locked copy of the raw data, one can refer to it to verify data integrity. To lock a file, go to File > Info > Protect Workbook > Always Open Read-Only. To begin analyzing the data, copy the data into a new workbook. For more precise control, Excel has additional protection levels in the Review tab. Functions such as Protect Sheet and Protect Workbook can be used to customize editing privileges.

## Tip 8: Use a file organization system to keep track of data as it is processed and analyzed

Now that your raw data file is securely saved in a read-only format, you can turn to working with your collected data—performing necessary tidying steps, calculating values to prepare for analysis, exploring the data to determine which statistical tests are appropriate, and statistically analyzing the values you measured during your experiment. For each of these steps, it is best to save your data in a new file. In the event that you make a mistake when working with the data (such as deleting something that you didn’t anticipate needing but finding out later you do need it), you will be able to go back to a prior or step or even your raw data and start again as needed.

Similarly, the creation of any finalized tables and figures that you intend to share or publish should be done in another file or sheet separate from the space used for cleaning your data. Having multiple tables with the same data or a spray of various figures clutters the analysis spreadsheet and may lead to the impression that those figures are fit for public consumption. And as we mentioned before, the steps used to format tables for the human eye often render the table unreadable to machines.

**Fig 4:**
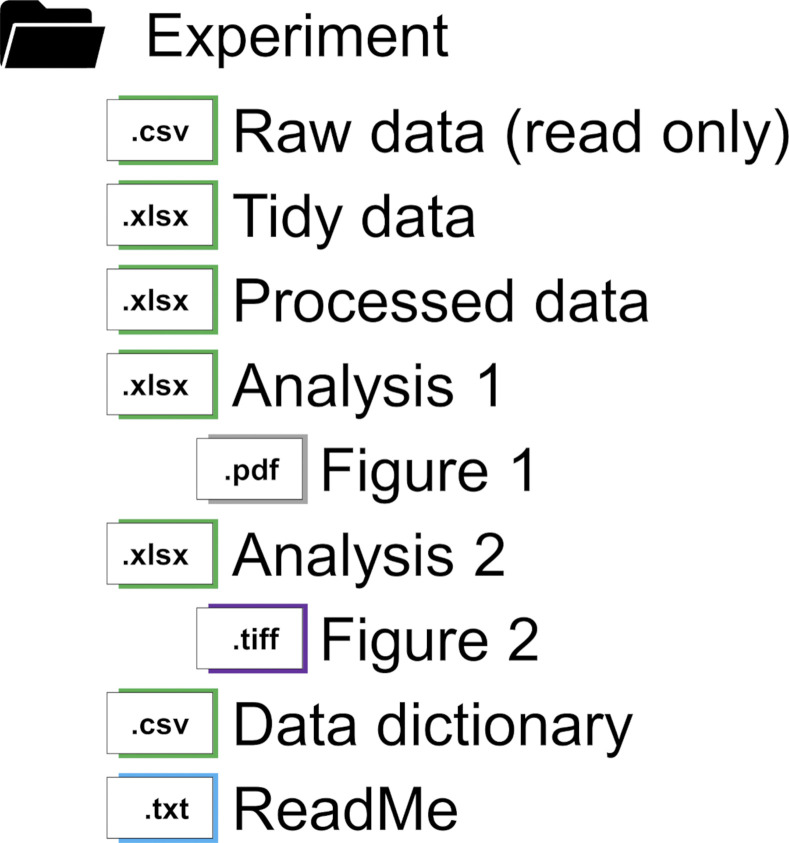
Example of a well-organized folder for an experiment which maintains separate files for each step in the research process. Metadata files include a data dictionary (see Fig 1A for an example) and a ReadMe, which is a plaintext file that compiles important information about the experiment. Note that the file icons displayed are not the only eligible format for that file type. For instance, metadata is commonly saved as a .txt file or use structure file formats such as XML or JSON.

The presence of multiple similarly named files can cause its own organizational problems. We recommend using a file organization system and naming convention so you can easily find the folders and files for raw data, processed data and final figures. Fig 4 demonstrates one acceptable organizational structure that houses the raw data, processed data and metadata in separate files. Note that the text after the file icons are short descriptions of the file context, not the file name.

## Tip 9: Include metadata in a separate file

Metadata is another important but often overlooked component of data curation and sharing. Metadata is structured information about data that facilitates its discovery, interpretation, and use. Think of metadata as all the notes you would make to yourself or to a colleague about the data. Metadata may also be automatically collected for some data types. For example, the properties of an Excel file on your computer will include metadata such as its format, location, size, and time of creation. Metadata should be documented in a machine-readable file format separate from the data. For the tardigrade example in Fig 1, the author should create a data dictionary which includes definitions for each of the measurements, the type of data for each measurement (string, number, date, etc.), and the acceptable values for each output (see the data validation parameters in Fig 3). Mayer and colleagues provides a short checklist to ensure your data dictionary is complete and machine readable [22]. For more detailed general information on data dictionaries, see Buchanan and colleagues [23], guidelines from Open Science Framework (OSF [24]), or project Open Data Metadata Schema [25]. It is also worthwhile to research the metadata standards for your field of study. For example, there are domain-specific data dictionaries for food allergy [26], brain injury [27], and cancer [28] among others. You can search the following registries for metadata schema related to your field of study: Research Data Alliance’s Metadata Standards Catalog [29], Fairsharing.org [30], Bioportal Ontologies [31], and the Digital Curation Centre guide to disciplinary metadata standards [32].

It is also good practice to keep a log of all cleaning steps and modifications of data over time. The Keep A Changelog resource includes tips for how to maintain an accurate manual changelog that prioritizes human readability [33]. This is especially important for collaborative projects when multiple people are accessing shared data. Ideally, one should be able to recreate a data analysis or data visualization using only the raw data and log file. Proper metadata documentation is an important function that supports the rigor, reproducibility, and transparency of research.

## Tip 10: Store files in non-proprietary formats

In the active research phase, we aren’t always thinking ahead to how someone 5 or 10 years in the future will be able to access the data. To maximize the usability of data and other artifacts of our work, it’s important to observe the FAIR Guiding Principles. First published in 2016, these principles state that good data management requires that digital objects be Findable, Accessible, Interoperable, and Reusable [1].

For long-term access files should be saved in non-proprietary formats—for example, you can save a text file as a .txt instead of a Microsoft Word document. This standard file type should be accessible using any word processing software, today, or many years in the future. While .xlsx is touted as an open file format, it is still best practice to preserve tabular data files in comma (.csv) or tab (.tsv) separated value formats. The US Library of Congress maintains a list of recommended formats for data sets [34]. Keep in mind that transferring files from one operating system to another—for example, a Windows machine to a MacOS machine—may disrupt formatting and integrity of your file. This is especially true with Microsoft products due to software version discrepancies between the 2 operating systems.

## Tip 11: Use the right tool for the job

We said this list of tips was going to focus on using Excel, but some projects warrant a move away from a “simple spreadsheet.” Luckily, there are a plethora of excellent resources to support you in taking that step. Each spreadsheet software has a hard limit as to the maximum number of rows and columns of data. While Excel sheets are limited to 1,048,576 rows and 16,384 columns per sheet, practically speaking, most data tables become too unwieldy long before reaching that threshold. Consider splitting large data tables into multiple tables or switching to a relational database management system (DBMS). A DBMS is software that allows users to store, retrieve, and run queries on data. It can be helpful to think about the system as many different blocks of data linked by common threads. Because all the data is not available in one spreadsheet, the user must ask the DBMS for the subset of data they need using a Structured Query Language (SQL). To upskill in DMBS, here is a sample of resources to get you started: R tutorials [35], introduction to SQL [36], and Data Carpentry courses for those who prefer live instruction [37]. For a scoping overview, we recommend the book *Data Management for Social Scientists* [38], which, despite its title, is broadly applicable to the analysis of tabular data and leads the reader from working with spreadsheets, to R, and finally to a DMBS. By learning new skills, you create an array of powerful possibilities for both data management, analysis, and visualization.

## Concluding remarks

Many researchers rely on Microsoft Excel due to its ubiquity and low barrier to entry. However, as pointed out throughout this review, typical usage of Excel and other similar spreadsheet software falls short in terms of data management and quality control. Just like a seasoned researcher would not be satisfied with the default chart options in Excel to graph their results, they should apply the same energy to maximizing the integrity of their tabular data in Excel throughout the research process. While these 11 quick tips have largely relied upon Excel-specific examples, these guidelines are broadly applicable to management of tabular data within any software.

As a final note, no researcher or data set is perfect. Improving your tabular data practice is a task you can take on one small step at a time and any improvement is worthwhile. Depending on the needs of your various projects, your approach to data management will evolve over time and may require customization on a per project or even per data type level. This is why preserving your raw data is so important—the way you analyze that data today will likely be different, perhaps less sophisticated, than the way you will analyze it months or years in the future. Build a culture of good data habits now to support the unforeseeable needs of you or other future users of your data.
